# Lessons learnt from COVID-19 in adult congenital heart patient in Tehran: a survey-based study of prevention, exposure, susceptibility, and outcomes

**DOI:** 10.1017/S1047951120004400

**Published:** 2020-11-18

**Authors:** Shabnam Mohammadzadeh, Ali Mehrakizadeh, Saeed Safari, Mohammadreza Mirzaaghayan, Roya Sattarzade Badkoubeh, Anahita Tavoosi, Akram Sardari, Saba Mohammadzadeh, Farnoosh Larti, Gruschen R Veldtman

**Affiliations:** 1 Department of Cardiology, Tehran University of Medical Sciences, Imam Khomeini Hospital Complex, Tehran, Iran; 2 Department of Cardiology, Children Medical Center, Tehran University of Medical Sciences, Tehran, Iran; 3 Department of Cardiology, Adult Congenital Heart Disease, King Faisal Specialist Hospital and Research Centre, Riyadh, Saudi Arabia

**Keywords:** COVID-19, adult congenital heart disease, questionnaire

## Abstract

**Background:**

COVD-19 pandemic has overwhelmed many healthcare systems worldwide. Underlying cardiovascular disease predisposes to greater disease susceptibility and more complications including mortality. Such data is unverified in adults with congenital heart disease (ACHD). The aim of the study is to report the Tehran experience with respect to preventative self-care measures, disease exposure, susceptibility, and outcomes after COVD-19 infection in ACHD patients.

**Methods:**

A telephone-based survey was conducted in ACHD patients, focusing on new-onset symptoms that might indicate COVID-19 infection, prevention measures, confirmed infection rates, and outcomes.

**Results:**

Three-hundred and nine ACHD patients, with a mean age of 29.13 years (range from 14 to 72 years, SD = 10.64), and 170 (55%) women were assessed. The majority (86.7%) had moderate or complex ACHD. Two-thirds (67.3%) of the patients practiced high-level preventative self-care measures. After community exposure, 33.3% developed COVID-19, and after household exposure, 43.7% developed COVID-19. There was only one mortality in a post-operative patient. Thirty-seven patients (12%) reported new symptoms including cough (10%), fatigue (8%), fever (7%), and new dyspnoea (6.5%). Amongst 18 (6%) with confirmed COVID-19, there was only 1 mortality in a post-operative patient. Age (adjusted OR = 1.19, 95% CI: 1.07–1.31, p = 0.001), contact with confirmed COVID-19 cases (adjusted OR = 59.34, 95% CI: 3.68–955.10, p = 0.004) were independently associated with COVID-19 infection.

**Conclusions:**

Mortality risk associated with COVID-19 infection in ACHD patients with moderate or severe disease appears to be relatively low, similar to the general population. Such risk appears to act through conventional risk factors, and in this cohort, we demonstrated age as a significant risk factor in addition to exposure to the development of COVID-19 infection. Preventative self-care measures are a potentially significant and impactful intervention target for intervention and for improving outcomes.

The COVID-19 pandemic has overwhelmed healthcare systems worldwide after its initial outbreak in the second half of 2019. Iran, a middle-income country, with a population of nearly 80 million was particularly hard hit by the pandemic, and official reports suggest 10,000 deaths and over 200,000 cases by June 2020.

Its underlying pathogen, novel beta coronavirus 2019, gains host cell entry utilising complex conformational change of its spike protein in response to binding with host cell membrane ACE-2 receptors, thereby enhancing its pathogenicity. Access to health care is vital for transforming outcomes particularly for those with moderate or severe disease. In Iran, health care is provided through three systems of care including the public–governmental sector which is the dominant system and is free to the public, the private sector, and non-governmental organizations founded by private citizens.^1^ Access to health care and its utilisation by patients, although varying by economic status, has generally been successfully established.

From clinical experience, approximately 20% of COVID-19 patients will require hospital admission, and in-hospital mortality has been as high as 25%. Patients with comorbidities are considered vulnerable to being infected as well as acquiring complications associated with infection. The same concerns exist for CHD as a potential predisposing risk factor,^2,3^ but to date, there is a paucity of data in ACHD populations. Routine screening of COVID-19 infection with viral RNA PCR techniques of patients is not considered feasible due to a large number of cases, resource shortage, and the temporal nature of test positivity necessitating testing on more than one occasion. Determining the prevalence of the disease based on patients’ symptoms alone is also challenging, as many patients are asymptomatic and multiple confounding factors may lead to an incorrect diagnosis of COVID-19 infection. Due to the lack of data in ACHD patients, and specifically, data from countries most severely affected by COVID-19 such as Iran, the present study was conducted to evaluate the prevalence of symptoms related to potential COVID-19 infection, the prevalence of confirmed cases, and to define the level of exposure and self-care measures in ACHD patients from Tehran, Iran.

## Material and methods

### Patients

Using the institutional patient database and electronic medical records of all ACHD patients seen in the private office of an ACHD cardiologist [SM] or seen in one of the tertiary centres in Tehran, Iran (Imam Khomeini Hospital Complex), potential study subjects were identified, and their contact details including telephone and addresses were documented.

### Inclusion criteria

The study protocol was approved by the Ethics Committee of Tehran University of Medical Sciences. Patients were included in the study if they had ACHD of moderate or severe disease complexity, and were assessed within the 1-year period prior to the study commencement. Patients with mild disease complexity were excluded from the study, except if they met the following criteria: (i) had a history of percutaneous interventional or surgical procedure during adulthood and/or (ii) had medical comorbidities (such as heart failure) or pulmonary hypertension.

### Study procedures

#### Assessment of baseline data

Detailed demographic, anatomic diagnoses, past medical and surgical history, functional class, physical examination at last visit, as well as latest laboratory and imaging data were reviewed and recorded. Measurement of oxygen saturation in room air using fingertip pulse oximetry done as a routine part of clinical examination was also recorded. A telephone-based survey was then conducted by two trained interviewers using a predefined questionnaire (see appendix) during the period from April 15, 2020 to April 30, 2020. The AHA/ACC classification of ACHD^4^ was used to categorise patients’ anatomical defects as being of mild, moderate, or severe complexity.

#### Assessment of new symptoms

Interviewers asked the patients regarding a recent change in Functional Class (FC) or new symptoms including the presence of any of the following symptoms: cough, fever, dyspnoea, myalgia, sore throat, and/or fatigue, during the time span from the first official announcement of COVID-19 infection in Iran up to the time of the telephone interview. Any hospital admissions during this time were also recorded. Additionally, patients were asked to report any prior laboratory confirmation of COVID-19 infections, and in the subsequent 14 days after the phone interview via a hotline that served as a connection with the patients. As the study population was from different cities with different social and familial supports, disease exposure was analysed with a detailed descriptive scale (appendix). Based on this descriptive scale, a newly developed classification for quantifying disease exposure and the practice of preventive self-care health behaviours against COVID-19 infection were used.

#### Assessment of preventative self-care measures amongst patients

For the purposes of this study, preventive self-care measures against COVID-19 were classified on five levels (Table 1). For sub-analysis, self-care levels 0 and 1 combined to make a new group of new “low self-care” level. Higher self-care levels (i.e., 2, 3, 4) were also combined to make a new “high self-care”.

**Table 1.** Summary of preventive self-care measures levels and disease exposure classification

Level 0	No specific measures are observed, i.e., no change in daily routine activity nor attitude
Level 1	Minimal self-care measures, including small changes in daily routine activity and attitude which means occasional handwashing and wearing a mask but not based precisely on WHO recommendations for the public^4^
Level 2	Restricted self-care measures, including protective measures based more accurately on WHO recommendations for the public, with adherence to social distancing protocols in the community
Level 3	Restricted self-care measures as noted in 2, and in addition adherence to governmental recommendations of staying at home and not going to work
Level 4	Restricted self-care measures as indicated in 3, and additionally applying such measures to all immediate family members, including social distancing and staying at home
**Disease exposure levels**	
0	No history of contact with symptomatic patient or confirmed COVID-19 patient
1	History of contact with symptomatic patient (suggestive of COVID-19 infection) or contact with definite COVID-19 case
2	Presence of confirmed COVID-19 case in the household member

#### COVID-19 exposure classification

We also divided patients into the following disease exposure categories. See Table 1. Occupational risk was also assessed telephonically both in the patient as well as in co-resident family members. High-risk careers were defined as healthcare providers and jobs that needed daily face-to-face interaction (e.g., healthcare system workers, working in a crowded area or close contact with a client).

### Statistics

Statistical analysis was performed using Statistical Package for the Social Sciences (SPSS) version 26.0 IBM®. Baseline data were described using descriptive tools including proportions and percentages for categorical data, means, and standard deviations (SD) for quantitative variables. Each quantitative variable was tested for normality and appropriate parametric or non-parametric tests were applied to compare differences in group characteristics. For qualitative variables, Chi-square test was used predominantly and for non-parametric variables, Mann–Whitney test was used. A p-value of ≤0.05 was considered significant. When the conditions for using Chi-square test were not fulfilled, we used Fisher’s exact test. Multivariate analysis by logistic regression was used to define independent determinants of outcomes.

## Results

### Baseline characteristics

**Figure 1.**
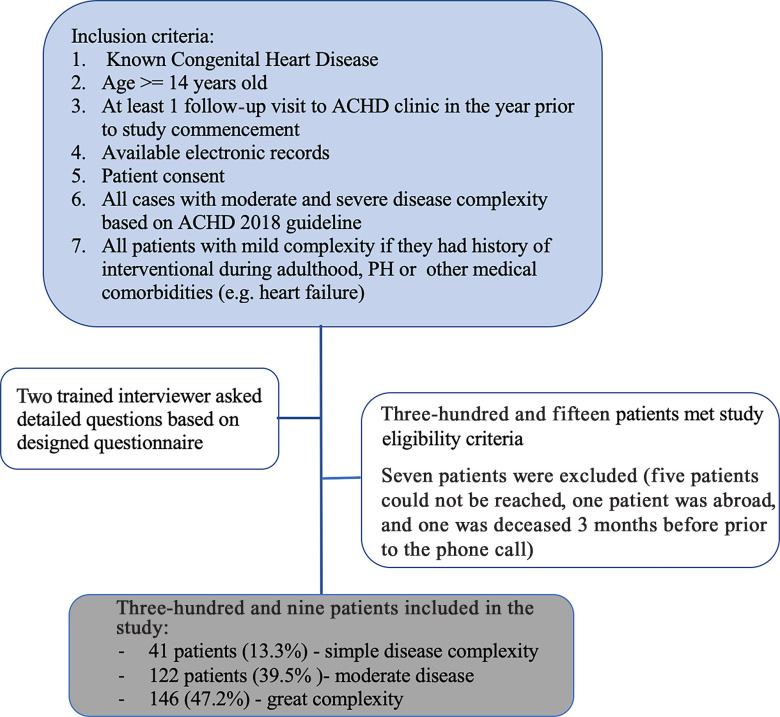
Summary of patient selection.

**Figure 2.**
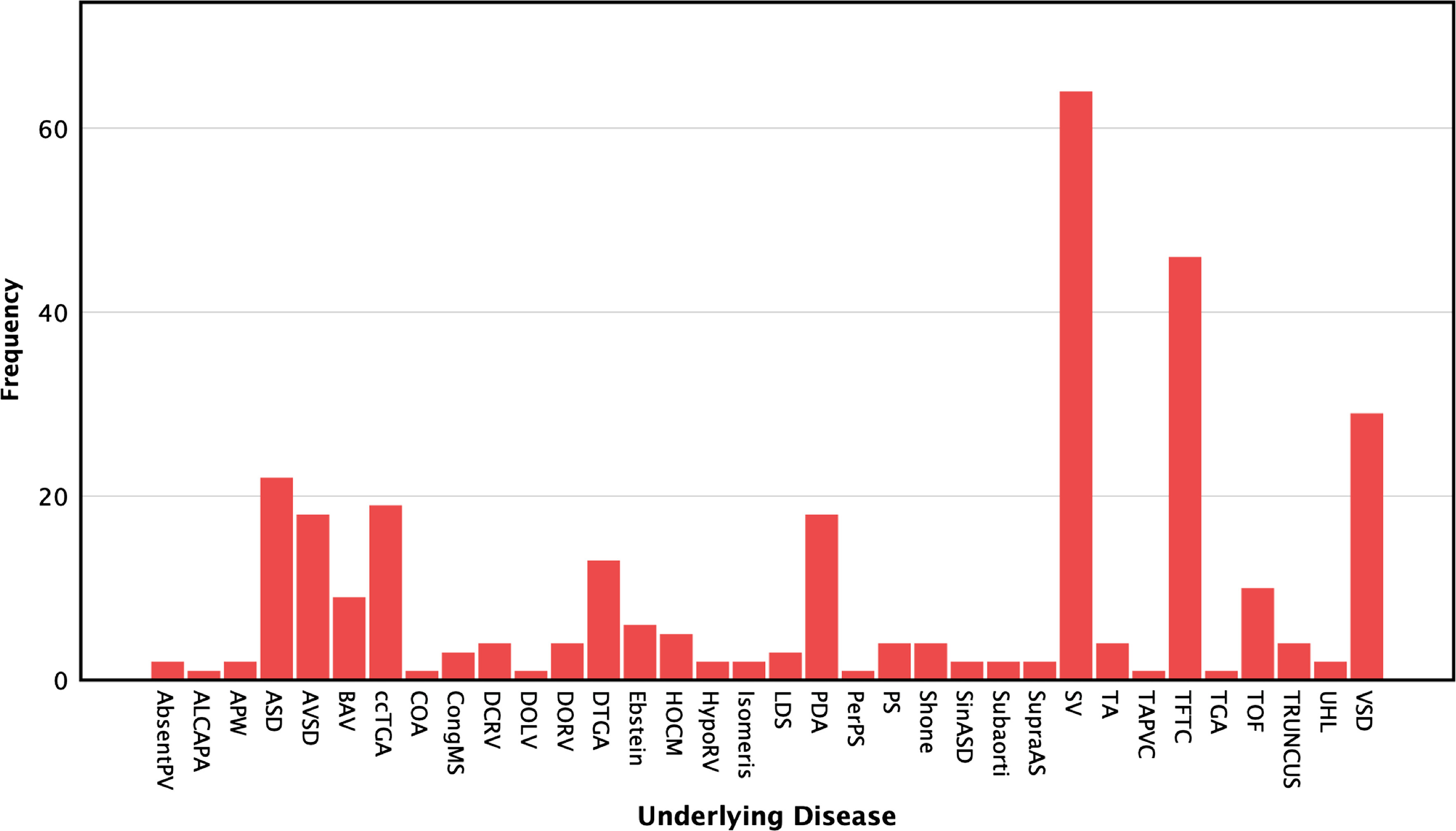
Frequency of underlying disease in the study population. Absent PV=Absent Pulmonary Valve; ALCAPA=Anomalous origin of Left Coronary Artery from Pulmonary Artery; APW=Aortopulmonary Window; ASD=Atrial Septal Defect; AVSD=Atrioventricular Septal Defect; BAV=Bicuspid Aortic Valve; ccTGA=Congenitally corrected Transposition of Great Arteries; COA=Coarctation of Aorta; CongMS=Congenital Mitral Stenosis; DCRV=Double Chamber Right Ventricle; DOLV=Double-Outlet Left Ventricle; DORV=Double-Outlet Right Ventricle; DTGA=Transposition of Great arteries; HOCM=Hypertrophic Cardiomyopathy; HypoRV=Hypoplastic RV; LDS=Loeys–Dietz Syndrome; PDA=Patent Ductus Arteriosus; PerPS=Peripheral Pulmonary Stenosis; PS=Pulmonary Stenosis; SinASD=Sinus venosus type ASD; Subaortic=Subaortic Stenosis; SupraAS=Supra-Aortic Stenosis; SV=Single Ventricle; TA=Tricuspid Atresia; TAPVC=Total Anomalus Pulmonary Vein Connection; TFTC=Tetralogy of Fallot after Total Correction; TGA=Transposition of Great Arteries; TOF=Tetralogy of Fallot; Truncus=Truncus Arteriosus; UHL=Uhl’s anomaly; VSD=Ventricular Septal Defect

Of the 315 patients who met the initially inclusion criteria, 7 patients were subsequently excluded. See Fig 1. A total of 309 patients, with a mean age of 29.13 years (range from 14 to 72 years, SD = 10.64), and 170 females (55%) were included in this survey. Based on the AHA/ACC ACHD classification, 41 patients (13.3%) had simple disease, 122 (39.5%) moderately complex disease, and 146 (47.2%) patients had greatly complex disease. Figure 2 demonstrates the frequency of underlying disease. In Table 2, the demographic data, ACHD AP classification, imaging and laboratory data, comorbidities, and drug history of the study population is denoted.

**Table 2.** Demographic data, ACHD AP classification, imaging, and laboratory data of the study group

Characteristics	Study group (n = 309) (%)
Age (years), mean (SD)	29.13 (10.64)
Sex, (%) women	170 (55%)
ACHD AP classification, n (%)	
Simple defect	41 (13.3%)
Moderate defect	122 (39.5%)
Complex defect	146 (47.2%)
Fontan patient, n (%)	61 (19.7%)
Eisenmenger syndrome, n (%)	33 (10.7%)
Type of surgery, n (%)	
Palliative	89 (28.8%)
Corrective	142 (46.0%)
No	78 (25.2%)
Percutaneous intervention, n (%)	
ASD closure	7 (2.3%)
PDA closure	4 (1.3%)
Coarctation angioplasty	2 (0.6%)
O_2_ saturation%, mean (SD)	92.01 (7.88)
Hgb, (n = 303, gr/dl), mean (SD)	15.06 (3.05)
Available data regarding PH condition, n (%)	227 (73.46%)
Echocardiographic estimation of mean PAP (n = 105), mmHg	34.56 (22.92)
Echocardiographic estimation of sPAP (n = 104), mmHg	30.02 (13.28)
Invasive measurement of mean PAP (n = 18), mmHg	36.72 (26.31)
Evidence of PH^*^	79 (25.6%)
Evidence of severe PH^†^	41 (13.3%)
NYHA functional class (n = 308), %	
I	119 (38.7%)
II	151 (49%)
III	38 (12.3%)
Systemic ventricle function, n (%)	
Normal	109 (35.3%)
Mildly impaired	142 (46.0%)
Moderately impaired	44 (14.2%)
Severely impaired	14 (4.5%)
Type of care, n (%)	
Self-care level 0	26 (8.4%)
Self-care level 1	75 (24.3%)
Self-care level 2	41 (13.3%)
Self-care level 3	103 (33.3%)
Self-care level 4	64 (20.7%)
Contact with confirmed COVID-19 cases, n (%)	
No contact	278 (90.0%)
Contact in the community	15 (4.8%)
Contact with the household members	16 (5.2%)
Contact with symptomatic patient (symptoms related to COVID-19), n (%)	
No	280 (90.6%)
Yes	29 (9.4%)
Confirmed COVID-19 cases, n (%)	
PCR	9 (2.9%)
Chest CT	6 (1.9%)
Both modalities	3 (1.0%)
Recent hospital admission, n (%)	
COVID-19 related	3 (1.0%)
Non-COVID-19 related	12 (3.9%)
High-risk job in the family, n (%)	
No	281 (90.9%)
Yes	28 (9.1%)
Symptoms, n (%)	
New cough	31 (10%)
New fatigue	25 (8.1%)
New sore throat	23 (7.4%)
Fever	22 (7.1%)
New dyspnoea	20 (6.5%)
New desaturation	5 (1.6%)
Comorbidities, n (%)	
Hypothyroidism	35 (11.3%)
Hepatic failure	9 (2.9%)
Interstitial lung disease	9 (2.9%)
Diabetes	6 (1.9%)
Renal failure	5 (1.6%)
PLE	4 (1.3%)
Hepatorenal	3 (1.0%)
Drugs, n (%)	
Vit D	232 (75.1%)
ASA	86 (27.8%)
Warfarin	80 (25.9%)
Betablocker	77 (24.9%)
ACEi/ARB	76 (24.6%)
PDE5 inhibitor	75 (24.3%)
Furosemide	42 (13.6%)
Bosentan	35 (11.3%)
Injection of influenza vaccine in the past year	18 (5.8%)

PLE=Protein-losing enteropathy; ACEi/ARBs=Angiotensin-converting enzyme inhibitor/Angiotensin receptor blocker; PDE5 inhibitor=Phosphodiesterase 5 inhibitor

* PH=Pulmonary hypertension (echocardiographic sPAP ≥ 38 mmHg or mean PAP ≥ 25 mmHg with echocardiography or catheterisation)

† Severe PH was considered present if echocardiographic sPAP ≥ 60 mmHg, or echocardiographic mean PAP or invasive mean PAP ≥ 40 mmHg

### Preventative self-care measures

Self-care measures are summarised in Table 2. Preventative self-care level was significantly associated with age, with patients <30 years of age being more likely to exhibit greater degrees of preventative self-care measures (p-value = 0.04). Patients with pulmonary hypertension and Eisenmenger syndrome were more likely to have 0–1 levels of self-care (low self-care). (p-value = 0.03 and 0.015, respectively) with no significant difference in symptoms or COVID-19 infection between in these high-risk groups regarding self-care level.

### COVID-19 exposure

**Figure 3.**
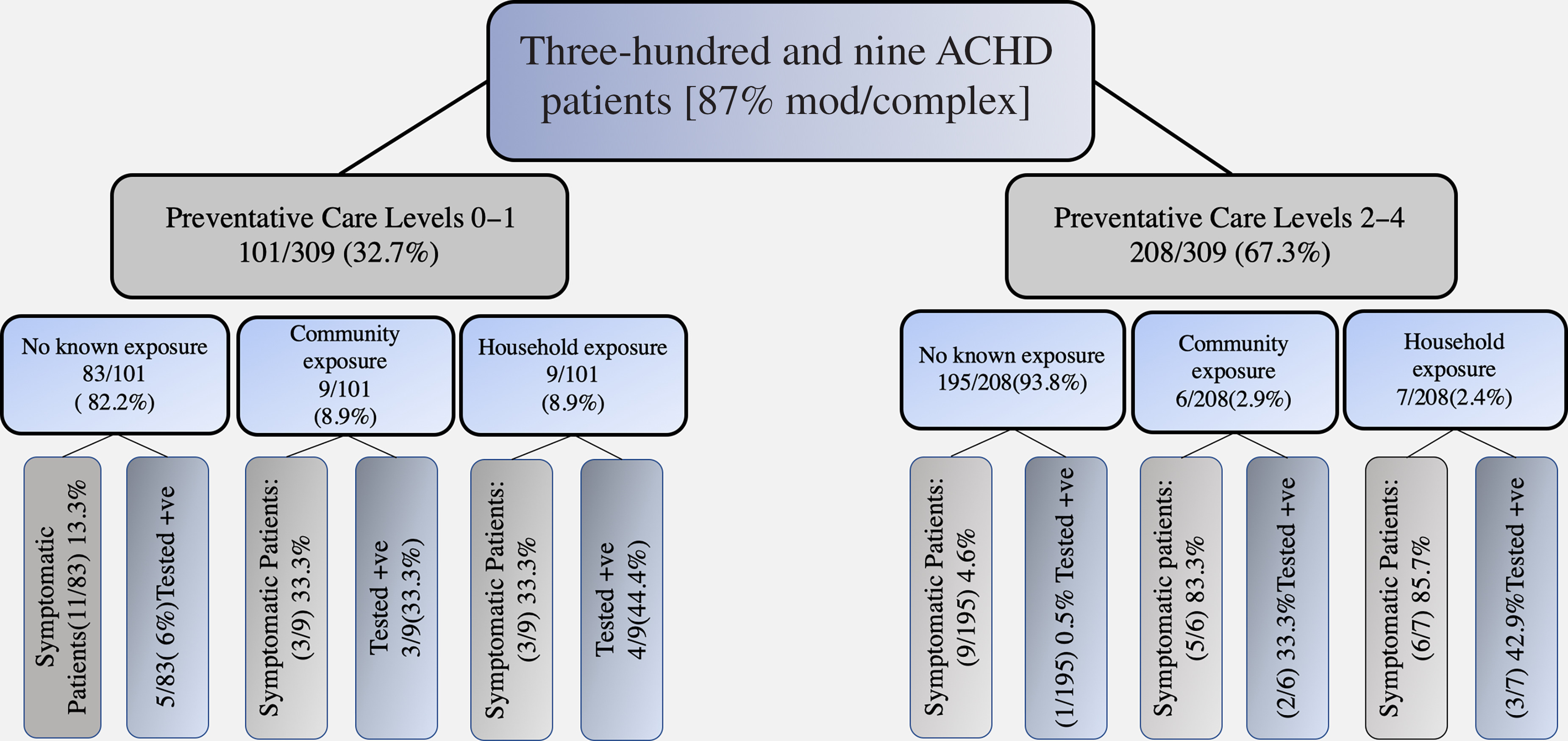
Frequency of symptomatic and confirmed COVID-19 patients amongst varying self-care levels and exposure levels. Two-thirds (67.3%) of the study population practiced high-level preventative self-care measures.

Fifteen patients (4.9%) reported a history of direct contact with a confirmed COVID-19-positive individual in the community. Of these, five (33.3%) were diagnosed with COVID-19 infection (two with the RNA polymerase test and three with chest CT). Sixteen patients (5.2%) reported contact with a COVID-19-positive family member living within the same household. Of these, seven (43.75%) were confirmed positive with COVID-19 infection (six with using the RNA polymerase test and one with chest CT). In Fig 3, the frequency of symptomatic and confirmed COVID-19 cases was shown in different exposure and self-care levels. In 28 patients (9.1%), either the patient or one of their family members occupied a high-risk job. Of these, four (14.28%) were confirmed positive (one with the RNA polymerase test, one with chest CT, and two with both methods). We found no significant difference in exposure level in different subgroups of the patients (i.e., Eisenmenger syndrome, Fontan patients, cyanotic patients, and patients with pulmonary hypertension).

### New symptoms indicating possible COVID-19 infection

Thirty-seven patients (11.9%) reported at least one new symptom including cough in 31/309 (10.0%), fatigue in 25/309 (8.1%), sore throat in 23/309 (7.4%), fever in 22/309 (7.1%), new dyspnoea in 20/309 (6.5%), and new desaturation (≥5%) in 1.6% (Fig 5a). Though symptomatic patients had similar demographic and clinical characteristics (see Table 3), they were significantly more likely to have had contact with a confirmed COVID-19 patient (p = 0.001, adjusted OR = 14.37, 95% CI: 3.63–56.88) and as well as contact with another symptomatic individual (p < 0.001, adjusted OR = 7.02, 95% CI: 1.84–26.72) (Fig 5b).

**Table 3.** Comparison of demographic and clinical data amongst symptomatic cases versus apparently asymptomatic cases

Characteristics	Symptomatic patient (n = 37)	Asymptomatic patient (n = 272)	p-value
Age (years), mean (SD)	35.05 (14.12)	28.32 (9.84)	0.06^*^
Sex, (%) women	23 (62.2%)	147 (54%)	0.35
ACHD AP classification, n (%)			
Simple defect	6 (16.2%)	35 (12.9%)	0.47
Moderate defect	17 (46%)	105 (38.6%)	
Complex defect	14 (37.8%)	132 (48.5%)	
Fontan Patient, n (%)	6 (16.2%)	55 (20.2%)	0.56
Eisenmenger syndrome, n (%)	3 (8.1%)	30 (11%)	0.58
Pulmonary-hypertension (n = 79), (%)	10 (34.5%)	69 (34.8%)	0.96
O_2_ saturation (%), mean (SD)	93.33 (7.25)	92.26 (7.51)	0.88
Hgb (n = 303, gr/dl), mean (SD)	14.01 (3.01)	15.01 (2.95)	0.37
Type of surgery, n (%)			
Palliative	12 (32.5%)	77 (28.3%)	0.77
Corrective	15 (40.5%)	127 (46.7%)	
No	10 (27%)	68 (25%)	
Percutaneous intervention, n (%)			
No intervention	35 (94.6 %)	261 (96%)	0.44
ASD closure	2 (5.4%)	5 (1.8%)	
PDA closure	0 (0%)	4 (1.5%)	
Coarctoplasty	0 (0%)	2 (0.7%)	
NYHA functional class (n = 308), (%)			
I	16 (43.2%)	103 (38%)	0.49^*^
II	12 (32.5%)	139 (51.3%)	
III	9 (24.3%)	29 (10.7%)	
Systemic ventricle function, n (%)			
Normal	12 (32.4%)	97 (35.7%)	0.53
Mildly impaired	15 (40.5%)	127 (46.7%)	
Moderately impaired	7 (19%)	37 (13.6%)	
Severely impaired	3 (8.1%)	11 (4%)	
Type of care, n (%)			
Self-care level 0	4 (10.8%)	22 (8.1%)	0.13
Self-care level 1	13 (35.1%)	62 (22.8%)	
Self-care level 2	7 (19%)	34 (12.5%)	
Self-care level 3	10 (27%)	93 (34.2%)	
Self-care level 4	3 (8.1%)	61 (22.4%)	
Contact with confirmed COVID-19 cases, n (%)			
No contact	20 (54.1%)	258 (94.8%)	0.001
Contact in the community	8 (21.6%)	7 (2.6%)	
Contact with the household members	9 (24.3%)	7 (2.6%)	
Contact with symptomatic patients (symptoms related to COVID-19), n (%)			
No	22 (59.5%)	258 (94.9%)	<0.001
Yes	15 (40.5%)	14 (5.1%)	
High-risk job in the family, n (%)			
No	30 (81.1%)	251 (92.3%)	0.059
Yes	7 (18.9%)	21 (7.7%)	
Comorbidities, n (%)			
Hypothyroidism	9 (24.3%)	26 (9.6%)	0.44^*^
Hepatic failure	2 (5.4%)	7 (2.6%)	0.29
Interstitial lung disease	2 (5.4%)	7 (2.6%)	0.29
Diabetes	1 (2.7%)	5 (1.8%)	0.53
Renal failure	3 (8.1%)	2 (0.7%)	0.60^*^
PLE	0 (0%)	4 (1.5%)	1.0
Hepatorenal	1 (2.7%)	2 (0.7%)	0.31
Drugs, n (%)			
Vit D	32 (86.5%)	200 (73.5%)	0.08
ASA	18 (48.6%)	68 (25%)	0.69^*^
Warfarin	14 (37.8%)	66 (24.3%)	0.07
Betablocker	12 (32.4%)	66 (24.3%)	0.39
ACEi/ARB	12 (32.4%)	64 (23.5%)	0.23
PDE5 inhibitor	8 (21.6%)	67 (24.6%)	0.68
Furosemide	14 (37.8%)	28 (10.3%)	0.051^*^
Bosentan	2 (5.4%)	33 (12.1%)	0.28
Injection of influenza vaccine in the past year	2 (5.4%)	16 (5.9%)	1.0

PLE: Protein-losing enteropathy; ACEi/ARBs: Angiotensin-converting enzyme inhibitor/Angiotensin receptor blocker; PDE5 inhibitor: Phosphodiesterase 5 inhibitor

* In preliminary univariate analysis, these factors were significant, after logistic regression, controlling for the effects of confounding factors, these individual factors did not maintain independent significance

### Confirmed COVID-19 cases

COVID-19 infection was confirmed in 18 patients (5.8%), with a mean age of 43.06 years (SD = 15.09). COVID-19 infection was confirmed by chest CT in six patients (1.9%), with real-time reverse transcriptase polymerase chain reaction (RT-PCR) in nine patients (2.9%) and with both modalities in three patients (1.0%). Ten patients (55.5%) of the definite confirmed COVID-19 group were female. Their underlying disease is summarised in Table 4 but in brief: simple in 3 (16.7%), moderate in 9 (50.0%), and complex in 6 (33.3 %).

**Table 4.** Comparison of demographic and clinical data amongst confirmed COVID-19 cases versus unknown status of COVID-19 infection

Characteristics	Confirmed COVID-19 cases (n = 18)	Unknown COVID-19 infection status (n = 291)	p-value
Age (years), mean (SD)	43.06 (15.09%)	28.27 (9.61%)	<0.001
Sex, (%) women	10 (55.5%)	160 (55.0%)	0.96
ACHD AP classification, n (%)			
Simple defect	3 (16.7%)	38 (13.1%)	0.47
Moderate defect	9 (50%)	113 (38.8%)	
Complex defect	6 (33.3%)	140 (48.1%)	
Fontan patient, n (%)	2 (11.1%)	59 (20.3%)	0.54
Eisenmenger syndrome, n (%)	2 (11.1%)	31 (10.7%)	1.00
Pulmonary hypertension (n = 79)	6 (42.9%)	73 (34.3%)	0.56
O_2_ saturation (%)	92,73 (5.14%)	92.36 (7.60%)	0.59
Hgb (n = 303, gr/dl), n (%)	14.7 (4.22%)	14.91 (2.89%)	0.68
Type of surgery, n (%)			
Palliative	6 (33.3%)	83 (28.5%)	0.52
Corrective	6 (33.3%)	136 (46.7%)	
No	6 (33.4%)	72 (24.8%)	
Percutaneous intervention, n (%)			
No intervention	17 (94.4%)	279 (95.8%)	0.73
ASD closure	1 (5.6%)	6 (2.1%)	
PDA closure	0 (0%)	4 (1.4%)	
Coarctoplasty	0 (0%)	2 (0.7%)	
NYHA functional class (n = 308), n (%)			
I	6 (33.3%)	113 (39%)	0.28^*^
II	6 (33.3%)	145 (50%)	
III	6 (33.4%)	32 (11%)	
Systemic ventricle function, n (%)			
Normal	5 (27.8%)	104 (35.7%)	0.52
Mildly impaired	8 (44.4%)	134 (46%)	
Moderately impaired	3 (16.7%)	41 (14.1%)	
Severely impaired	2 (11.1%)	12 (4.2%)	
Type of care, n (%)			
Self-care level 0	3 (16.7%)	23 (7.9%)	0.08^*^
Self-care level 1	9 (50%)	66 (22.7%)	
Self-care level 2	2 (11.1%)	39 (13.4%)	
Self-care level 3	3 (16.7%)	100 (34.4%)	
Self-care level 4	1 (5.5%)	63 (21.6%)	
Contact with confirmed COVID-19 cases, n (%)			
No contact	6 (33.3%)	272 (93.5%)	0.00
Contact in the community	5 (27.8%)	10 (3.4%)	
Contact with the household members	7 (38.9%)	9 (3.1%)	
Contact with symptomatic patient (symptoms related to COVID-19), n (%)			
No	12 (66.7%)	268 (92.1%)	0.23^*^
Yes	6 (33.3%)	23 (7.9%)	
High-risk job in the family, n (%)			
No	14 (77.8%)	267 (91.8%)	0.06
Yes	4 (22.2%)	24 (8.2%)	
Symptoms, n (%)			
New cough	16 (88.9%)	15 (5.2%)	<0.001
New fatigue	15 (83.3%)	10 (3.4%)	<0.001
New sore throat	11 (61.1%)	12 (4.1%)	<0.001
New dyspnoea	14 (77.8%)	6 (2.1%)	<0.001
Fever	13 (72.2%)	9 (3.1%)	<0.001
New desaturation	4 (22.2%)	1 (0.3%)	<0.001
Comorbidities, n (%)			
Hypothyroidism	3 (16.7%)	32 (11%)	0.44
Hepatic failure	0 (0%)	9 (3.1%)	1.00
Interstitial lung disease	1 (5.6%)	8 (2.7%)	0.42
Diabetes	0 (0%)	6 (2.1%)	1.00
Renal failure	2 (11.1%)	3 (1%)	0.56^*^
PLE	0 (0%)	4 (1.4%)	1.00
Hepatorenal	1 (5.6%)	2 (0.7%)	0.16
Drugs, n (%)			
Vit D	15 (83.3%)	217 (76.4%)	0.57
ASA	9 (50%)	77 (26.5%)	0.42^*^
Warfarin	4 (22.2%)	76 (21.6%)	1.0
Betablockers	5 (27.8%)	73 (25%)	0.79
ACEi/ARB	5 (27.8%)	71 (24.4%)	0.77
PDE5 inhibitor	4 (22.2%)	71 (24.4%)	1.00
Furosemide	6 (33.3%)	36 (12.4%)	0.27^*^
Bosentan	1 (5.4%)	34 (11.7%)	0.70
Injection of Influenza Vaccine in the past year	3 (16.7%)	15 (5.2%)	0.07

PLE=Protein-losing enteropathy; ACEi/ARBs=Angiotensin-converting enzyme inhibitor/Angiotensin receptor blocker; PDE5 inhibitor=Phosphodiesterase 5 inhibitor

* In preliminary analysis, p-values were ≤0.05 but after logistic regression for controlling the effects of confounding factors, p-values did not reach the level of significance

**Figure 4.**
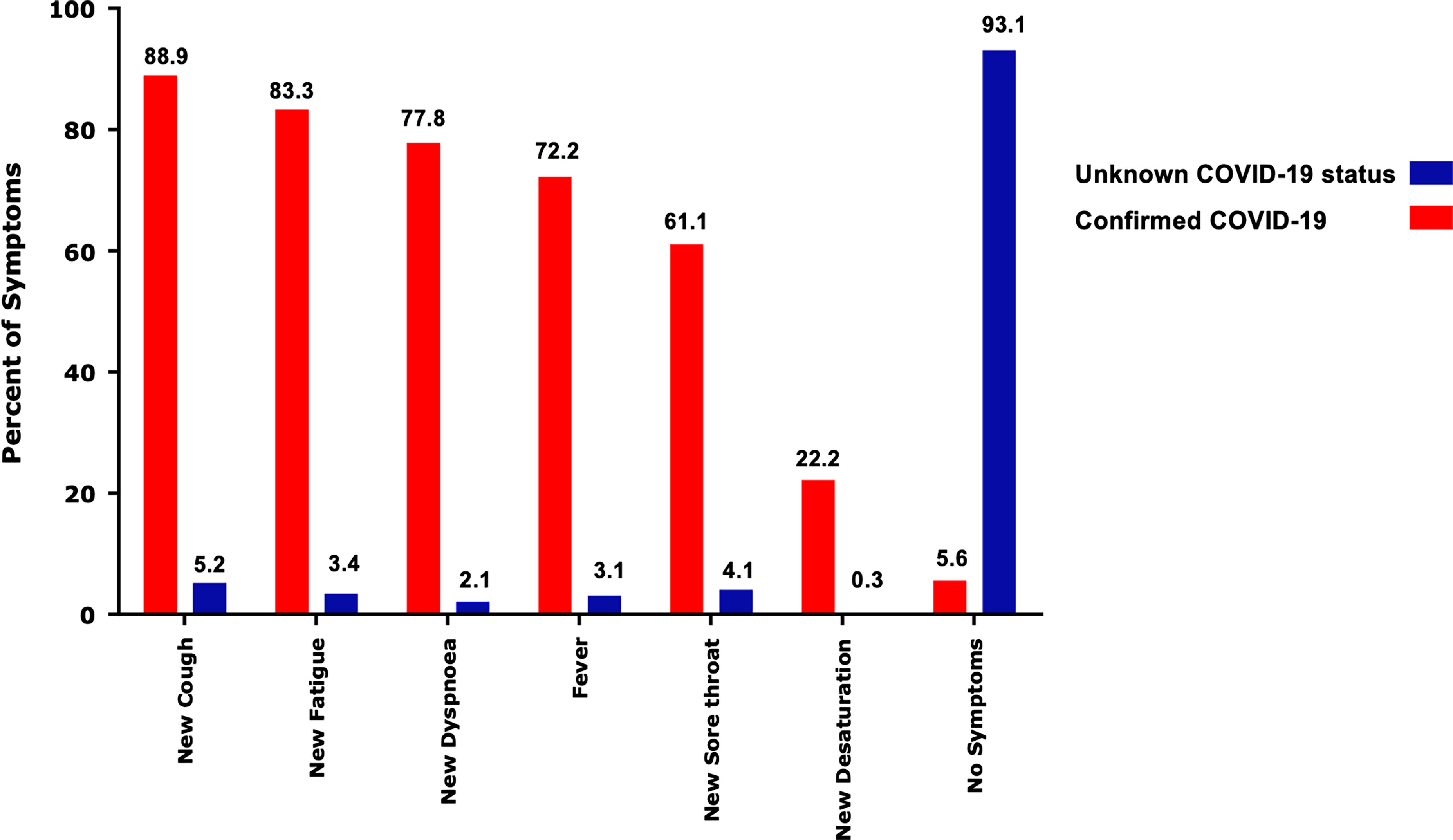
Frequency of symptoms in confirmed COVID-19 patients compared with patients with unknown COVID-19 status. Cough, fatigue, and dyspnoea were the three most common in affected patients; only one confirmed COVID-19 patient was asymptomatic.

Seventeen patients (94.44%) of the 18 confirmed COVID-19-positive patients were symptomatic with mild disease. The most common new symptoms were cough in 16/18 patients (88.9%), fatigue in 15/18 cases (83.3%), dyspnoea and change in FC in 14/18 (77.8%), fever in 13/18 patients (72.2%), and sore throat in 11/18 patients (61.1%) (Fig 4). In 15 of 18 (83.33%) infected patients, more than two new symptoms (i.e., fatigue, dyspnoea, cough, sore throat, fever, and desaturation more than 5%) were reported. Only one patient with COVID-19 disease did not report any symptoms: a 65-year-old man with a large ventricular septal defect (VSD) and severe PH who did not adhere to any preventative self-care measures (self-care level 0) with confirmed COVID-19 disease in one of his household members. Compared to patients with unknown disease status, confirmed cases of COVID-19 disease were older (mean age 43.06, SD = 15.09 26. versus mean age 28.27, SD = 9.71 with p ≤ 0.001). Indeed, for each year of age, the risk of COVID-19 infection increased by 1.19 times (adjusted OR =  1.19, 95% CI: 1.07–1.31). Confirmed COVID-19 ACHD patients were also significantly more likely to have had a history of contact with a confirmed COVID-19 individual (adjusted OR = 59.34, 95% CI: 3.68–955.10, p = 0.004). Further, confirmed COVID-19 infection in ACHD patients showed a significant association with new-onset symptoms. (p < 0.001). Combining new symptoms and confirmed COVID-19 cases, the prevalence of suspected and confirmed COVID-19 infection was significantly higher in patients ≥30 years old. (p-value = 0.004 and 0.001, respectively).

### Outcomes

Moderate or severe symptoms necessitated hospital admission in only two cases (5.4%) of 37 symptomatic patients. Only 1/18 confirmed COVID-19 patients (5%) died. He was a 54-year-old man, with Ebstein’s anomaly of the tricuspid valve who was admitted for and underwent a first redo tricuspid valve replacement for intractable right-sided heart failure, i.e., initial admission was non-COVID-19 related. Screening for COVID-19 infection was not performed before the surgery as he was asymptomatic from this standpoint. After surgery, he had an eventful intensive care unit course due to localised tamponade and hypotension. After surgical drainage of the effusion, no improvement in clinical symptoms was noticed and respiratory symptoms complicated the post-surgical course. He subsequently tested positive for COVID-19. He had shared an ICU room with another patient that also subsequently tested positive for COVID-19.

**Figure 5.**
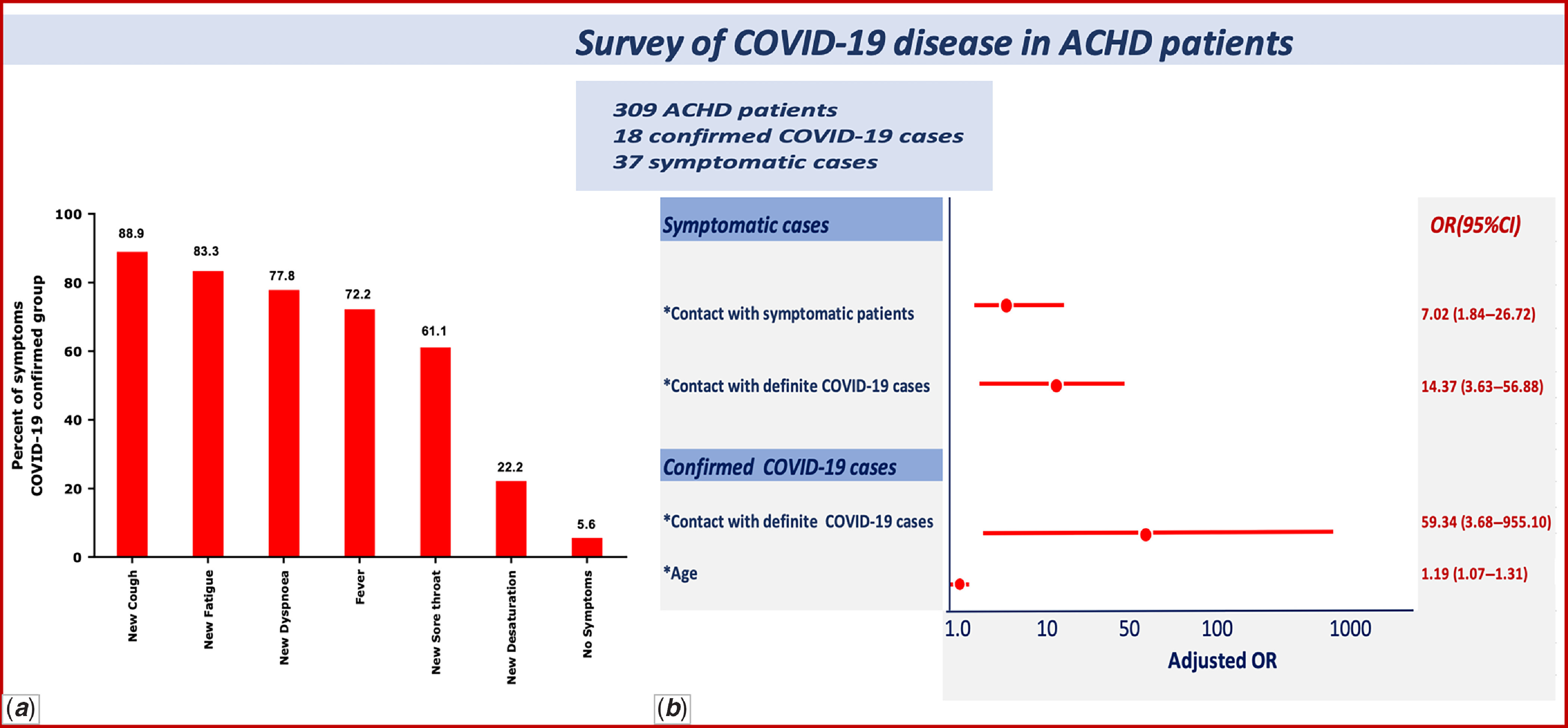
Brief summary of study results. (***a***) Frequency of symptoms in confirmed COVID-19 cases. (***b***) Adjusted OR (95% CI) of statistically significant variables in symptomatic and confirmed COVID-19 patients.

In Table 5, the prevalence of symptoms, definite COVID-19 disease, level of self-care, and exposure were compared in different subgroups of ACHD patients that are usually considered high risk. Graphical summary of the study is presented in Fig 5.

**Table 5.** Comparison of symptoms, definite COVID-19 disease, level of self-care, and exposure in high-risk subgroups of ACHD patients

	Confirmed COVID-19	Unknown COVID-19 status	p-value	Symptomatic patients	Asymptomatic patients	p-value	Low self-care	High self-care	p-value
Fontan Non-Fontan	2 (3.3%) 16 (6.5%)	59 (96.7%) 232 (93.5%)	0.542	6 (9.8%) 31 (12.5%)	55 (90.2%) 217 (87.5%)	0.566	18 (29.5%) 83 (33.5%)	43 (70.5%) 165 (66.5%)	0.55
PH No PH	6 (7.6%) 8 (5.4%)	73 (92.4%) 140 (94.6%)	0.567	10 (12.7%) 19 (12.8%)	69 (87.3%) 129 (87.2%)	0.969	33 (41.5%) 41 (27.7%)	46 (58.2%) 107 (72.3%)	0.03
Eisenmenger Non-Eisenmenger	2 (6.1%) 16 (5.8%)	31 (93.9%) 260 (94.2%)	1.00	3 (9.1%) 34 (12.3%)	30 (90.9%) 242 (87.7%)	0.78	17 (51.5%) 84 (30.4%)	16 (48.5%) 192 (69.6%)	0.015
Normal EF Reduced EF	13 (5.2%) 5 (8.6%)	238 (94.8%) 53 (91.4%)	0.348	27 (10.8%) 10 (17.2%)	224 (89.2%) 48 (82.8%)	0.17	76 (30.3%) 25 (43.1%)	175 (69.7%) 33 (56.9%)	0.061
Diuretics No diuretics	6 (14.3%) 12 (4.5%)	36 (85.7%) 255 (95.5%)	0.023	14 (33.3%) 23 (8.6%)	28 (66.7%) 244 (91.4%)	<0.001	16 (38.1%) 85 (31.8%)	26 (61.9%) 182 (68.2%)	0.42
Age <30 Age ≥30	3 (1.7%) 15 (11.5%)	175 (98.3%) 116 (88.5%)	<0.001	13 (7.3%) 24 (18.3%)	165 (92.7%) 107 (81.7%)	0.003	50 (28.1%) 51 (38.9%)	128 (71.9%) 80 (61.1%)	0.04

PH=pulmonary hypertension; EF=Ejection fraction of the systemic ventricle

## Discussion

Despite ACHD being a potentially important underlying comorbidity predisposing to acquiring COVID-19 infection and its adverse outcomes, data regarding COVID-19 infection in this patient group is striking in its paucity.^5^ To our knowledge, this is the first publication not only reporting an extended case series of ACHD patients with COVID-19 infection, and examining the impact of preventive self-care measures, infection exposure, disease susceptibility, and outcomes, but also to document the experience with such patients in Tehran, that saw some of the gravest impacts of the COVID-19 pandemic. The key observations of this study were that almost two-thirds of the patients with confirmed infection had documented community or household exposure to a known infected individual, and that such individuals were older than those without COVID-19 infection. Generally, symptoms were mild and only one death was documented. We found significant room for improving on preventative self-care measures such as social distancing, hand washing, and masking, particularly in households with an infected individual.

Gatzoulis^6^ in a recent editorial stressed the role and importance of patient education in preventing COVID-19 infection in ACHD patients. Preventive measures are probably one of the most important and effective strategies in the fight against the high transmissibility of COVID-19. In the present series, one-third of the patients failed to adhere to adequate preventative self-care measures. Intriguingly, younger patients were more likely to practice good preventive self-care measures. Important health behaviours such as these vary according to geographic location, educational level, cultural, and sociopolitical factors. Other key influencers are factors related to illness perception.^7^ Our data suggest a crucial need for systematic educational programmes targeting particularly middle and older aged individuals with CHD.

About 44% of patients developed confirmed infection after household exposure, and a near 33% developed COVID-19 positivity after community exposure to an infected individual. Older age, and the presence of new-onset symptoms such as cough, fever, dyspneoa, myalgia, sore throat, and/or fatigue were strong and independent predictors of testing positive for COVID-19. The WHO estimated the reproductive number [R0] of COVID-19 being between 1.4 and 2.5, meaning that each infected individual is likely to transmit the disease from 1.4 to 2.5 other individuals on average, setting the stage for widespread population dissemination of pandemic proportions.^8^ Older patients, those with obesity, hypertension, diabetes, and the immunocompromised, appear to have a greater susceptibility to acquiring COVID-19.

Most of the infections in this series were apparently mild and only one mortality was documented in a post-operative patient with initially undetected COVID-19. Severity of the underlying CHD did not appear to play an important role in COVID-19 disease severity. The generalisability of these initial observations is unclear at this stage and we advise continued vigilance and surveillance for new symptoms, in especially those indicating moderate or severe COVID-19 disease. Case fatality rate in the general population has been estimated at <1% for those < 50 years old, 1.3%50–60 years, 3.6% for 60–80 years old, and 14.8% for octogenarians. Pre-existing cardiovascular disease represents one of the greatest case fatality risk factors with an average 10.5% mortality, relative to the 7.3% for diabetes, 6.3% for chronic respiratory disease, 6.0% for hypertension, and 5.6% for cancer.^9^ In the present series, the single mortality that occurred in a patient admitted for surgery and with COVID-19 infection that was probably acquired in the hospital, is a clear warning that hospital infection control systems require inpatient surveillance of COVID-19 infection, especially in high-risk patient groups.

In this report, 37 of the 309 patients, i.e., approximately 10% of those surveyed had become symptomatic, and 54% of these patients did not seek any medical attention, suggesting mostly minor symptoms. The prevalence of COVID-19 infection in the general population varies considerably by country and region. When systematic screening is employed, prevalences of as high as 18%^10^ have been documented. Such systematic data of COVID-19 infection did not yet exist regionally at the time of this study. Of some surprise to us, ACHD disease severity classification, systemic ventricular function, SaO_2_, Hgb level, FC, and type of care did not seem to differ between symptomatic versus asymptomatic patients in this study. These early observations suggest that CHD anatomy per se does not confer greater risk. In our study, an ejection fraction of the systemic ventricle, functional class, pathologies such as PLE, or the presence of other comorbid conditions had no association with COVID-19 infection. Supplemental vitamin D was prescribed in 75.1% of the study population. We were not able to examine the impact of vitamin D in this population, though prior studies have suggested potential benefits.^11^ We caution against adopting complacency and encourage data collection in wider registries to better define the epidemiologic and clinical characteristics of COVID-19 in adults with CHD.

## Conclusions

COVID-19 infection risks do not appear to be exaggerated in patients with moderate or severe CHD. Risk of acquiring the infection appears to be similar to the general population, and we document in this presented age as an important risk factor in addition to household exposure. Meaningful preventative self-care measures should be addressed through education and focus on measured associated with better disease prevention such as social distancing, masking, and handwashing. In these preliminary observations, CHD severity did not seem to predispose to greater COVID-19 susceptibility, nor to more complications or higher mortality outcomes. Indeed, it is our belief that COVID-19 infection exerts its risk through conventional cardiovascular and other risk factors such as heart failure, diabetes, hypertension, diabetes, immunocompromise, and obesity. This needs verification in larger observational studies such as currently being undertaken. Recognising that our findings of relatively high-quality preventative self-care strategies being practiced by ACHD patients in this particular cohort, may have strong cultural and local sociopolitical influences, we strongly advocate for continued vigilance and systematic surveillance for COVID-19 in ACHD patients, and advise clinicians to urgently create educational programmes aimed at enhancing knowledge of COVID-19. Such education programmes should emphasise those at risk, identifying symptoms that indicate deterioration such as shortness of breath, worsening cyanosis, and also the practice of high-quality preventative self-care measures.

## Study limitations

Though the present study represents one of the largest published series of COVID-19 infection in adults with CHD, the overall numbers were still too small to make broader statistical inferences. We did not perform any systematic serological, imaging, or PCR test in asymptomatic patients, and it is possible that we missed some asymptomatic-positive patients. As this study was based on a telephonic interview, it was by definition dependent on accurate recall, and accuracy of subject reporting. Additionally, it is likely that there was considerable overlap in the levels of self-care and disease exposure not defined by the predetermined levels used in this investigation. Patients were asked to call the ACHD_COVID-19 hotline if they developed symptoms within 14 days of the initial telephone contact, and it is possible that some patients may not have reported new symptoms, or new hospital admissions, leading to a potential underestimation of COVID-19 infection in this report. The classification of preventative self-care behaviours, and the classification of disease exposure was developed specifically for the purpose of this study and was not specifically validated in wider populations. However, the relative simplicity of the measure helps to mitigate concerns regarding its validity. There is also a very significant likelihood that COVID-19 infection may be under-represented in this study, similar to other published series. This is due to the underlying rate of asymptomatic infection which is not always known for a particular population.
